# Transient Corneal Response After Goldmann Three-Mirror Gonioscopy and Its Limited Effect on Optical Biometry and Calculated Intraocular Lens Power: A Prospective Within-Subject Controlled Observational Study

**DOI:** 10.3390/medicina62081576

**Published:** 2026-08-17

**Authors:** Ahmet Taner Uysal, Ahmet Mehmet Somuncu, Adem Türk

**Affiliations:** 1 Department of Ophthalmology, Trabzon Kanuni Training and Research Hospital, 61290 Trabzon, Türkiye; mehmetsomuncu89@hotmail.com; 2 Department of Ophthalmology, Faculty of Medicine, Karadeniz Technical University, 61080 Trabzon, Türkiye

**Keywords:** Goldmann gonioscopy, optical biometry, cataract surgery, intraocular lens power, central corneal thickness, equivalence testing, repeated measures, difference in differences

## Abstract

*Background and Objectives*: Contact gonioscopy and optical biometry often happen at the same preoperative visit, with no defined interval between them. We asked whether uncomplicated Goldmann three-mirror gonioscopy was followed by short-term changes in optical biometry and calculated intraocular lens (IOL) power and whether any mean change could be positively affirmed as small enough not to matter clinically. *Materials and Methods*: Sixty-four cataract patients (128 eyes) underwent NIDEK AL-Scan biometry at baseline and 5, 15 and 30 min after unilateral Goldmann three-mirror gonioscopy in this prospective repeated-measures study (2015–2016). The untreated fellow eye served as an internal control. Our primary analysis was a within-patient paired difference-in-differences (DiD) comparison, which modeled the paired-eye structure directly; nested patient/eye mixed-effects models followed as a sensitivity analysis. We recomputed IOL power as a continuous variable for four formulas, SRK/T, Haigis, Hoffer Q and Holladay 1, using the published constants for the implanted lens. Equivalence within ±0.25 D was tested by two one-sided tests (TOSTs), with Holm adjustment across the twelve tests performed. *Results*: Central corneal thickness (CCT) rose by 3.14 µm at 5 min (95% CI, 1.94 to 4.35; *p* < 0.001) and by 1.66 µm at 15 min (*p* = 0.015), returning to baseline by 30 min. That signal sat inside the ±6.2 µm short-term repeatability seen in untreated control eyes. Keratometry moved by no more than 0.11 D, and all four IOL formulas behaved almost identically, the largest mean difference being −0.18 D at 15 min. Equivalence within ±0.25 D was confirmed in unadjusted testing at 5 and 30 min for all four formulas and at 15 min for SRK/T; after Holm adjustment only the four 30 min conclusions and the 5 min conclusion for SRK/T were retained. The selected 0.5 D lens power changed in 33–47% of study eyes and in a comparable 31–52% of untreated control eyes. *Conclusions*: Procedure-related differences in calculated IOL power were small throughout. The largest was −0.18 D, inside the ±0.25 D margin and well below the 0.5 D interval on which lenses are supplied. Demonstrated equivalence was a narrower claim. After adjustment for multiplicity, it held at 30 min for all four formulas and at 5 min for SRK/T, and at 15 min it was not demonstrated for Haigis, Hoffer Q or Holladay 1. These data therefore do not establish that biometry is unaffected across the whole first half hour, and we make no recommendation about deferring it. The transient CCT signal fell within device repeatability. Step-level changes in selected lens power were as common in eyes that never underwent gonioscopy. All analyses were exploratory and limited to a partial-coherence-class biometer of the study period and to the four formulas tested.

## 1. Introduction

Cataract surgery is now planned as a refractive procedure, and refractive accuracy afterwards rests on reliable preoperative biometry and on transparent reporting of how intraocular lens (IOL) power was arrived at [1,2,3,4]. Optical biometers measure axial length (AL), corneal curvature, anterior chamber depth (ACD), central corneal thickness (CCT), pupil size and white-to-white corneal diameter (WTW) repeatably, and each of these feeds into the power estimate either directly or through the effective lens position [2,3,4,5,6,7,8]. Precision, device repeatability, the state of the ocular surface and short-term biologic or technical drift can all move the numbers the biometer returns [9,10,11,12,13,14].

The preoperative work-up usually adds other examinations, some of them contact procedures. Gonioscopy is one that has not been displaced: it is still how the anterior chamber angle is assessed in glaucoma, pseudoexfoliation syndrome, ocular hypertension, narrow angles and pigment dispersion. Anterior-segment imaging has grown more available, but clinical gonioscopy remains the reference because it shows the angle directly and allows dynamic assessment [15,16,17].

The question here is one of workflow rather than physiology. In a busy cataract clinic the preoperative visit is a single appointment: the patient is examined, the angle is assessed where that is indicated, and biometry is acquired in the same session. Which of the two comes first often depends on room availability and patient flow rather than on any protocol. Sending the patient back on another day is always possible, but it costs them an attendance and the clinic a slot, and it is worth doing only if the measurement is genuinely disturbed. Thus, the question is not whether one could wait, it is whether one must, and for how long. Answering it means measuring the size of the disturbance and how quickly it goes away.

Pupil dilation deserves a word. Gonioscopy comes before any mydriatic, and none was given at any point during the observation window here. We report pupil diameter among the endpoints so that this can be checked rather than taken on trust. The sequence studied is gonioscopy followed by biometry in an undilated eye, which is what routine practice looks like.

Goldmann three-mirror gonioscopy requires topical anesthesia, coupling gel and direct contact between the diagnostic lens and the cornea. Any of these can redistribute the tear film, leave a brief residual gel layer, change epithelial hydration or introduce minor mechanical effects. A biometer may read such surface phenomena as an apparent corneal change even when the stroma is untouched. The clinically useful question is not whether optical biometry can detect these changes, but whether they are ever large enough to alter IOL power selection.

Earlier work directly examining gonioscopy-related corneal measurement effects is sparse. George et al. reported transient corneal curvature and topographic changes after gonioscopy, particularly during the first 5 to 15 min, and emphasized that the effect of contact examinations may be relevant when keratometry is performed immediately afterwards [18]. Related work on same-day contact or near-contact ophthalmic measurements supports the broader principle that the ocular surface can show short-term measurement variability after instrumentation [19]. Other short-term biometric studies have tended to be limited—smaller samples, younger or mixed populations, fewer endpoints, or no clinically anchored IOL power measure.

We set out to close part of that gap by pairing CCT with a formally tested IOL power endpoint across four formulas, so that clinical relevance rather than statistical significance decides what counts. The present study used a within-subject fellow-eye control schedule with within-patient paired analysis. Because the eye undergoing gonioscopy was chosen by clinical indication rather than randomization, the study is framed as a prospective controlled observational study. We hypothesized that uncomplicated Goldmann three-mirror gonioscopy would produce a small transient corneal measurement signal without a clinically meaningful change in calculated IOL power. Because the data were acquired in 2015–2016 with a partial-coherence-class optical biometer, the findings are presented as period-specific and are not assumed to transfer directly to contemporary swept-source biometers or to formula suites released since.

## 2. Materials and Methods

### 2.1. Study Design and Ethics

This prospective, repeated-measures, within-subject controlled observational study was conducted at the Department of Ophthalmology of the Trabzon training hospital affiliated at the time with Karadeniz Technical University Faculty of Medicine, Trabzon, Türkiye, between July 2015 and July 2016. The institution was subsequently reorganized under Trabzon University, which accounts for the difference between the current author affiliation and the institution named for the data-collection period.

The study was conducted in accordance with the Declaration of Helsinki (1975, revised in 2013) and was approved by the Ethics Committee of Karadeniz Technical University Faculty of Medicine (Approval No. 2015/66; approval date: 8 June 2015). Written informed consent was obtained from all participants prior to enrollment. Reporting followed the STROBE framework where applicable.

All 64 patients were recruited consecutively within the single window stated above, between July 2015 and July 2016, under Approval No. 2015/66. Sixty of them also appeared in the residency thesis of the first author [20]. The thesis dataset was closed at the first 60 consecutive patients, following the supervisor’s instruction that the thesis statistics be based on that number; the four patients recruited last—the final four of the consecutive series, examined on 23 June 2016 (one patient), 1 July 2016 (two patients) and 5 July 2016 (one patient), as the recruitment window closed—were therefore not carried into the thesis analysis and enter the published record for the first time here. They were not recruited after the thesis, and the statement to that effect in the previous version was an error, which we correct here: it described when those four records entered the analysis dataset, not when the patients were seen. Ref. [20] is dated 2019 because that is when the thesis was written and defended, several years after data collection ended. The four patients followed the same protocol, in the same center, with the same device and the same examiner as the rest of the cohort, and gave written informed consent on the same form. Because no recruitment took place outside the approved window, their inclusion falls under the original approval, and no separate or supplementary ethics approval was sought or required. A sensitivity analysis restricted to the 60 thesis patients is reported in Section 3.6 and shows no change in direction or interpretation of any result.

Because the eye undergoing gonioscopy was determined by routine clinical indication rather than random allocation, the investigation should be regarded as a prospective controlled observational study rather than a randomized paired-eye trial. The fellow eye served only as a short-term untreated measurement reference during the 30 min observation window; the design did not imply absence of clinical indication in the fellow eye, and routine clinical care was not withheld because of study participation.

### 2.2. Study Population and Eligibility Criteria

We recruited consecutive patients scheduled for routine phacoemulsification with IOL implantation who also needed preoperative gonioscopy. One eye per patient underwent Goldmann three-mirror gonioscopy and became the study eye. The fellow eye went through the identical measurement schedule untouched, giving us an internal estimate of how much repeated biometry drifted on its own.

Inclusion criteria were age-related cataract requiring phacoemulsification, age 50 years or older, clinical indication for gonioscopy, ability to maintain stable fixation, high-quality optical biometry and written informed consent. Exclusion criteria were previous ocular surgery or laser treatment, corneal ectasia or dystrophy, clinically significant corneal edema, active ocular inflammation, infectious keratitis, severe ocular surface disease, recent contact lens wear, ocular trauma, media opacity preventing reliable optical biometry, poor fixation, or inability to complete repeated measurements.

### 2.3. Statistical Framework, Sample Size and Clinical Relevance Threshold

What follows is an analysis of prospectively collected data, not a pre-registered trial. We wrote no prospective statistical analysis plan, performed no a priori sample size calculation, and settled on CCT as the primary endpoint during the analysis rather than before collection began. The endpoint hierarchy and multiplicity strategy set out below are therefore retrospective and exploratory. No result reported here should be read as confirmatory. We say so plainly. Presenting a post hoc hierarchy as though it had been prespecified would misrepresent how strong the evidence is.

A retrospective power calculation built on the observed effect would add nothing, so we report the precision the study actually achieved. The acquisition protocol helps here: every value entering the analysis is the mean of three separate measurements, so what follows describes averages rather than single readings. Across 64 patient pairs the half-width of the 95% confidence interval for a within-patient DiD estimate was ±1.29 µm for CCT, ±0.096 D for mean keratometry, ±0.110 D for SRK/T power and ±0.139 D for Haigis power. The smallest true difference the study could detect with 80% power was 1.81 µm for CCT and 0.15–0.20 D for calculated IOL power. The study is therefore adequately precise to resolve differences well below the ±0.25 D equivalence margin, which is the relevant question for the equivalence claim. Inferences about the absence of a clinically meaningful effect are made only where equivalence was formally demonstrated.

A mean calculated IOL power difference smaller than ±0.25 D was predefined as clinically negligible. Routine IOL powers are supplied and selected in 0.5 D increments, so an average shift below half of that interval cannot systematically change lens choice. At the spectacle plane, the same 0.25 D shrinks further, to roughly 0.17 D for an average eye at a 1.5 ratio. That is below the level at which most patients notice a refractive difference, and below the smallest step in which spectacles are routinely dispensed. The margin is therefore conservative from both a lens-selection and a refractive-outcome standpoint [3,4].

### 2.4. Gonioscopy Procedure

One experienced examiner performed every gonioscopy, following a standardized protocol. After 0.5% proparacaine hydrochloride, sterile coupling gel went onto the contact lens, and the examination proceeded under dim illumination with the patient in primary gaze. Excessive corneal compression, inadvertent indentation, repeated repositioning of the lens and prolonged contact were all avoided. Each examination took roughly two minutes.

The coupling agent was Viscotears liquid gel (carbomer 980, 2 mg/g; Dr. Gerhard Mann Chem.-Pharm. Fabrik GmbH, Berlin, Germany), applied to the concave surface of the diagnostic lens in a volume sufficient to fill it to the rim of the lens sphere. After removal of the gonioscopy lens the ocular surface was irrigated, and the lens was cleaned and re-charged with gel before the next examination. All post-procedure time points were timed from removal of the gonioscopy lens rather than from the start of the examination, so the first acquisition followed lens removal by 5 min.

We chose 5, 15 and 30 min to bracket the interval over which contact-related corneal change had previously been reported to appear and fade. George et al. found transient curvature and topographic change concentrated in the first 5 to 15 min after gonioscopy [18], and tear-film and epithelial recovery after contact procedures is usually described over much the same span. Five minutes was meant to catch the peak, 15 the decay, 30 the return towards baseline. No published protocol prescribes a schedule for this question, so the choice is pragmatic and we present it as such.

Anterior chamber angle configuration in the gonioscopy-treated eye was graded according to the Shaffer classification [16]; because the Shaffer scale is ordinal, grades are summarized as the median with range. No gonioscopy of any kind was performed on the fellow eye during the study, so no Shaffer grade was recorded for it; angle grades in this report describe study eyes only. This preserved the fellow eye as an untreated short-term measurement reference but limits inference about inter-eye anterior-segment comparability.

### 2.5. Optical Biometry and IOL Power Calculation

We used the NIDEK AL-Scan optical biometer (NIDEK Co., Ltd., Gamagori, Japan), measuring at baseline before gonioscopy and again 5, 15 and 30 min afterwards. Recorded variables were K1 and K2 at the 2.4 mm and 3.3 mm optical zones, CCT, ACD, AL, WTW, pupil diameter and calculated IOL power [21,22]. K1 and K2 denote the flatter and steeper keratometric powers. Their arithmetic mean, Km, is the keratometric variable behind every model reported here. Best-corrected visual acuity (BCVA) was taken as decimal Snellen equivalent and served for baseline description only.

Powers were calculated for emmetropia. Patients received the Sensar AR40e three-piece hydrophobic acrylic IOL (Abbott Medical Optics, Santa Ana, CA, USA), and the biometer carried the published optical constants for that lens. We did not personalize them. Since the configuration governs everything that follows, we examined it after the fact by back-calculating from the stored outputs. With A = 118.7 and the 2.4 mm keratometric zone, the SRK/T algorithm reproduced 97.7% of the 511 checkable stored SRK/T values exactly after rounding to the 0.5 D grid—12 disagreed, every one of them by exactly one 0.5 D step, which was the largest deviation in the set. On the 3.3 mm zone the same calculation reproduced only 70.3%, and no more than 74.6% even after re-optimizing the A-constant for that zone. With the published Haigis triplet for that lens (a0 = −2.420, a1 = 0.157, a2 = 0.288), the Haigis algorithm reproduced 96.7% of the 510 checkable stored Haigis values exactly, again with every disagreement confined to a single 0.5 D step, against 63.5% on the 3.3 mm zone. The checkable totals were 511 and 510 rather than 512 because the two out-of-range entries described in Section 2.7 were treated as missing. A distinction is worth drawing here. The constants themselves are documented: A = 118.7 and the Haigis triplet are the published optical constants for the Sensar AR40e and were not derived from these data. What was inferred was the keratometric zone the device applied to its own calculation. We did not have access to a device configuration log, and the 2.4 mm assignment rested on the reproduction rates given above rather than on a service record. It should be read as a back-calculated inference, well supported by 97.7% and 96.7% exact agreement with a maximum deviation of one 0.5 D step, but an inference nonetheless.

One feature of the stored data shaped the whole analysis: the IOL powers the device recorded were already rounded to the 0.5 D grid on which lenses were supplied. Analyzing rounded values pushes quantization noise into the mean-level estimates, and it can manufacture differences between formulas that share the same inputs. We therefore recomputed IOL power as a continuous, unrounded variable from the measured AL, Km and ACD at every time point, using the verified constants. Four formulas were computed: SRK/T [21], Haigis [22], Hoffer Q [23] and Holladay 1 [5]. Hoffer Q and Holladay 1 derive the effective lens position from corneal power. We added them so that formulas with an explicit keratometric term would sit alongside Haigis, which uses anterior chamber depth for that purpose instead. The published lens constants for the Sensar AR40e were used throughout: A = 118.7 for SRK/T, the Haigis triplet given above, a personalized ACD of 5.41 for Hoffer Q and a surgeon factor of 1.63 for Holladay 1. Only the individual-eye lens-step analysis kept the rounded values, since there, the 0.5 D grid was itself the quantity of clinical interest.

Three separate acquisitions were taken for each eye and time point, and their arithmetic mean entered the dataset. Every value analyzed was therefore an average of three readings rather than a single one. Study and control eyes followed the identical sequence at every time point. The AL-Scan aligns and acquires automatically, but positioning, fixation quality and acceptance of a reading still depend on the operator, and the examiner knew which eye was which.

### 2.6. Outcome Measures

Longitudinal change in CCT after Goldmann three-mirror gonioscopy was the endpoint designated as primary during analysis. As Section 2.3 states, that designation came after the data rather than before. Secondary endpoints were mean keratometry at both optical zones, AL, ACD, WTW, pupil diameter and calculated IOL power by each of the four formulas. Clinically we wanted two things: whether any post-gonioscopy mean shift in IOL power could be affirmed as lying inside the predefined ±0.25 D margin, and how often the lens power that would actually have been ordered changed.

### 2.7. Statistical Analysis

The primary analysis was a within-patient paired difference-in-differences comparison. For each patient and each post-baseline time point we computed the change from baseline in the study eye, the change from baseline in the fellow control eye, and the difference between the two, then tested that difference against zero with a one-sample paired t test. Twenty-six of the 33 difference distributions departed from normality on Shapiro–Wilk testing, mostly through heavy tails contributed by a handful of eyes with unstable individual readings. Every primary comparison was therefore repeated with the Wilcoxon signed-rank test. Section 3.6 reports how far the two agree. The paired estimator used each patient as their own control. Correlation between the two eyes of one patient was removed by construction rather than modeled, and nothing needed be assumed about the between-eye covariance structure. All reported DiD estimates, confidence intervals and equivalence tests come from it.

Mixed-effects models served two purposes here: a sensitivity analysis, and omnibus tests of the group-by-time interaction. We used a two-level structure, with a random intercept for patient and a variance component for eye nested within patient, so that both sources of correlation appeared explicitly. One correction is owed to the submitted version, where we wrote that a single eye-level random intercept was preferred because patient-level variance was near zero. That was wrong, and the sentence has been removed. Patient-level variance was in fact substantial, and larger than the eye-within-patient component: for CCT, 750.1 µm^2^ at patient level against 37.6 µm^2^ for eye within patient, with a residual variance of 6.1 µm^2^. Both specifications nevertheless gave identical interaction test statistics, χ^2^ = 29.95 either way for CCT, because the group-by-time contrast was estimated within-eye and was orthogonal to the between-patient and between-eye intercepts. We report the nested specification throughout.

Omnibus interactions were tested by likelihood-ratio comparison of models with and without the interaction term, under maximum likelihood, with Holm adjustment across the ten secondary tests. Post-baseline pairwise DiD estimates carry no multiplicity adjustment and are explicitly exploratory. We give the unadjusted values so that readers can apply whichever correction they consider right; the Holm-adjusted equivalents appear in Supplementary Table S1.

For clinical equivalence we applied the two-one-sided-test (TOST) procedure to the within-patient DiD estimate of calculated IOL power at each post-baseline time point, taking ±0.25 D as the margin and declaring equivalence when the 90% confidence interval lay entirely inside it. A TOST verdict is a threshold decision, so we also asked how stable each verdict was, repeating the analysis with one patient omitted at a time and recording how often it reversed. Twelve TOST analyses were run in all, four formulas by three time points. No principal formula or time point was designated in advance, because no prospective analysis plan existed, and we did not designate one retrospectively: any such choice would be made among results already seen. Every equivalence conclusion in this report is therefore exploratory, and none is confirmatory. An earlier version argued that correlation between the tests, together with the conservatism of adjusting an equivalence test, meant an unadjusted analysis could not inflate the equivalence claim. That argument was not sound and has been withdrawn. The conservatism of TOST is a property of the individual test rather than of the family, and correlation reduces but does not remove the probability that at least one of twelve equivalence conclusions is spurious. We therefore report the unadjusted TOST *p* values, so that each comparison remains visible, alongside Holm-adjusted *p* values across the full family of twelve and draw all conclusions from the adjusted column. Failure to demonstrate equivalence is reported as such and is not interpreted as evidence of a clinically important difference.

At the individual-eye level, clinical impact was read off the device-stored powers, which already sit on the 0.5 D supply grid. Any non-zero change there is a change in the lens that would have been ordered, so the proportions in Section 3.5 are exactly that and not a proxy for it. Study and untreated fellow control eyes were compared by a paired McNemar test. We expected these comparisons to have limited power, and their interpretation rests mainly on how closely the study- and control-eye rates track each other.

Two isolated transcription errors surfaced during analysis, both in control eyes at the 15 min time point. One eye had K1 at the 2.4 mm zone recorded as 5.42 D, while the same eye measured 45.36, 45.30 and 45.24 D at the other three points. A second had ACD recorded as 0.12 mm against 3.13, 3.20 and 3.12 mm. In each case the leading digit was missing. A corneal power of 5.42 D and an anterior chamber depth of 0.12 mm fall outside the range the AL-Scan can return, so the device cannot have produced them. That argument establishes that these entries are not measurements. It does not establish what the true values were. An earlier version replaced them with 45.42 D and 3.12 mm, reconstructed from the missing leading digit; that reconstruction has been withdrawn. In the analysis reported here both entries are treated as missing, and each patient contributes to a comparison only where every value entering it is present. This leaves CCT unaffected at *n* = 64 throughout; mean keratometry, SRK/T, Hoffer Q and Holladay 1 fall to *n* = 63 at 15 min, and Haigis, which uses measured anterior chamber depth, to *n* = 62. Section 3.6 reports the alternative handlings—the reconstructed values, and exclusion of both patients from the cohort—as sensitivity analyses; all three agree on every estimate to within 0.006 D and on every equivalence verdict. Nothing else in the dataset was altered. Neither entry has been checked against a source document. The original AL-Scan printouts and paper data-collection forms are held in the hospital archive and are retrievable through the institution’s records procedure, but that check had not been made when this analysis was run, and until it has, we reconstruct nothing. Dataset S1 carries both the analysis dataset and an as_recorded sheet preserving every value exactly as originally entered, so the two entries can be inspected in their original form. Supplementary Note S1 reports every estimate and every equivalence verdict under all three handlings. An outlier screen was then applied across every measurement. Its thresholds were chosen at the analysis stage, after the data had been seen. It flagged any value that deviated from the median of that eye’s three other time points by more than was physiologically plausible over half an hour: 20 µm for CCT, 1.0 D for mean keratometry, 0.10 mm for axial length, 0.20 mm for ACD, 0.50 mm for white-to-white and 2.5 mm for pupil diameter. Flagged values all lay within the range the device could return, so we kept them as measured and quantified their influence by the sensitivity analysis in Section 3.6 rather than by correcting anything. The screen is reported as a sensitivity analysis only, and no inference about the cause of any flagged movement is drawn from it.

Residual and quantile–quantile plots were inspected for model diagnostics. Analyses ran on scripted Python (version 3.12) workflows using pandas (version 3.0), NumPy (version 2.4), SciPy (version 1.17), statsmodels (version 0.14) and Matplotlib (version 3.10). The anonymized dataset and the complete analysis code are provided as Supplementary Materials.

## 3. Results

### 3.1. Study Population

Sixty-four consecutively recruited eligible patients (128 eyes) entered the study, each contributing one eye for gonioscopy and one untreated fellow eye as internal control. Every patient completed the full sequence at baseline and at 5, 15 and 30 min. None were excluded after enrollment, and none withdrew during the short observation window. We did not keep a formal screening log of patients assessed but not enrolled, which we note as a reporting limitation.

The mean age was 69.44 ± 9.79 years (range 46–93); 32 patients were female and 32 male; the study eye was the right eye in 35 patients and the left in 29. Shaffer grade in study eyes was 2 in four eyes (6.2%), 3 in 31 (48.4%) and 4 in 29 (45.3%), with a median of 3 (range 2–4). Baseline characteristics are reported separately for study and control eyes in Table 1. As expected from clinically indicated, non-randomized allocation, BCVA was substantially lower in study eyes. The parameters that drive IOL power calculation—CCT, keratometry at both zones and axial length—did not differ between the two groups. Small differences were present in ACD (0.07 mm) and WTW (0.11 mm); both are an order of magnitude below any clinically relevant threshold and, given the sensitivity of the Haigis formula to ACD (0.29 D per mm), the ACD difference corresponds to 0.02 D of calculated power. Baseline calculated IOL power did not differ between groups by either formula.

### 3.2. Omnibus Interaction Tests

CCT showed a significant group-by-time interaction in the nested model (χ^2^ = 29.95, df = 3, *p* < 0.001). Among the secondary endpoints, all four IOL formulas reached significance after Holm adjustment with almost identical test statistics (χ^2^ = 12.87 to 13.06; Holm-adjusted *p* = 0.045 for each), and so did mean keratometry at the 3.3 mm zone. At 2.4 mm, keratometry did not survive adjustment. Nothing procedure-related emerged for AL, ACD, WTW or pupil diameter (Table 2).

That the four test statistics land so close together is itself informative. In the submitted version, computed from the device-rounded outputs, Haigis reached significance and SRK/T did not, and we read the dissociation as residual measurement variation propagating differently through the two formulas. Recomputing power as a continuous variable removed it entirely. All four formulas now move together, in the same direction and by similar amounts. The earlier discrepancy belonged to the 0.5 D quantization, not to the formulas. It follows that formula choice does not materially change what this study concludes, at least among formulas whose effective-lens-position term rests on keratometry, axial length or anterior chamber depth.

### 3.3. Central Corneal Thickness

**Figure 1:**
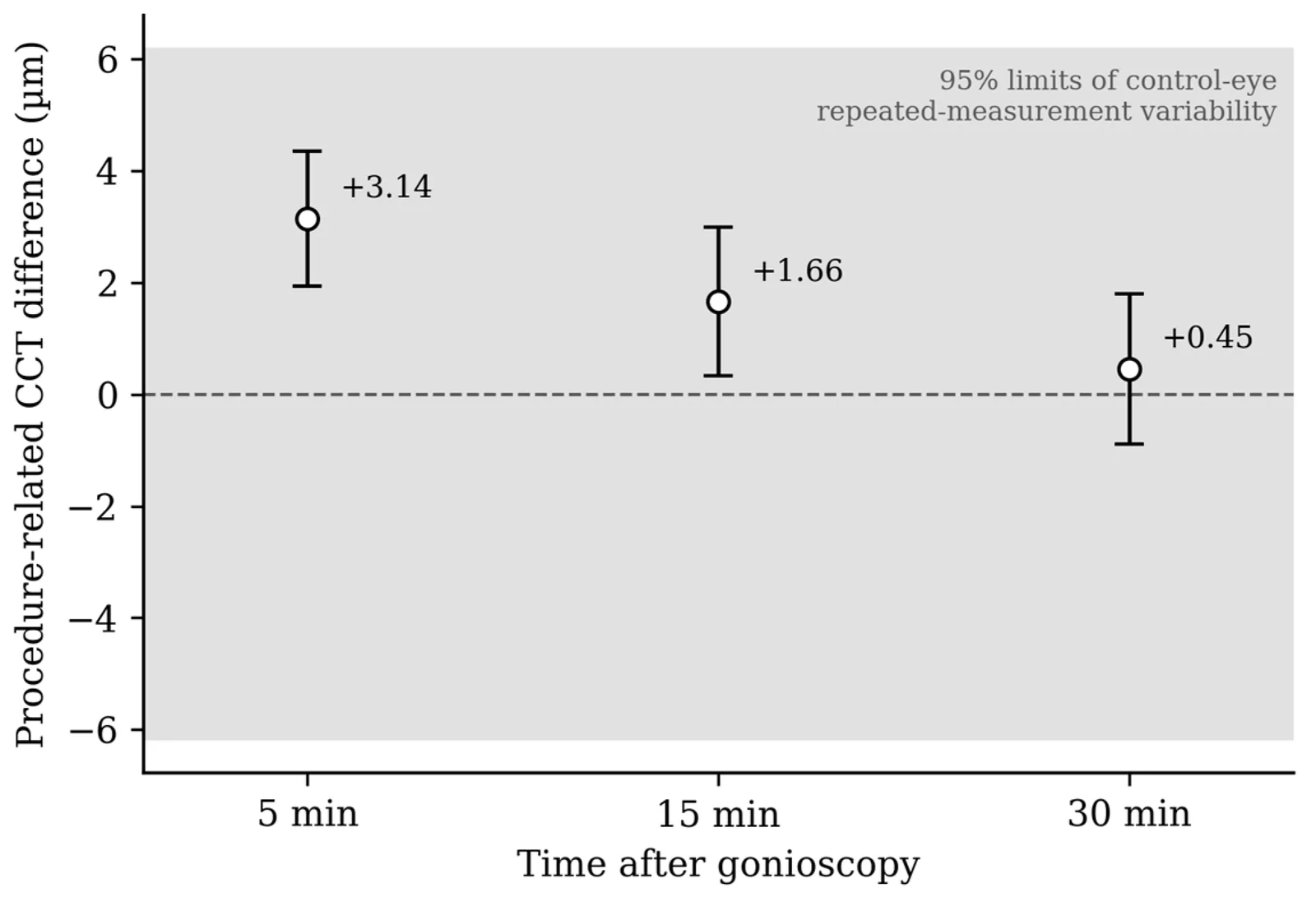
Procedure-related central corneal thickness (CCT) effect estimated by within-patient paired difference in differences (additional change in study eyes relative to the fellow control eye of the same patient) at 5, 15 and 30 min. Points are mean estimates; error bars indicate 95% confidence intervals; the dashed line marks no procedure-related difference.

Within-patient paired analysis showed an additional procedure-related CCT increase of +3.14 µm at 5 min (95% CI, +1.94 to +4.35; *p* < 0.001) and +1.66 µm at 15 min (95% CI, +0.33 to +2.98; *p* = 0.015). By 30 min the estimate had fallen to +0.45 µm and was no longer distinguishable from zero (95% CI, −0.90 to +1.80; *p* = 0.505) (Table 3; Figure 1).

How large is that? The right comparison is the background variability of the measurement itself. In the untreated fellow eyes, which underwent the identical measurement schedule without gonioscopy, CCT changed from baseline by 0.70 ± 3.14 µm across the three post-baseline time points, giving 95% limits of ±6.2 µm for repeated measurement over that interval, and since each value is the mean of three acquisitions, the corresponding limits for a single reading would be wider still. Published test–retest repeatability for CCT on the AL-Scan is of comparable magnitude, approximately 5.1 µm [11]. The +3.14 µm peak attributable to gonioscopy therefore lies inside the band within which a single repeated measurement of the same eye would be expected to vary in the absence of any procedure. The effect is real in the sense that it is reproducible across 64 paired eyes and would not arise by chance; it is not detectable in an individual patient.

### 3.4. Keratometry and Calculated IOL Power

**Figure 2:**
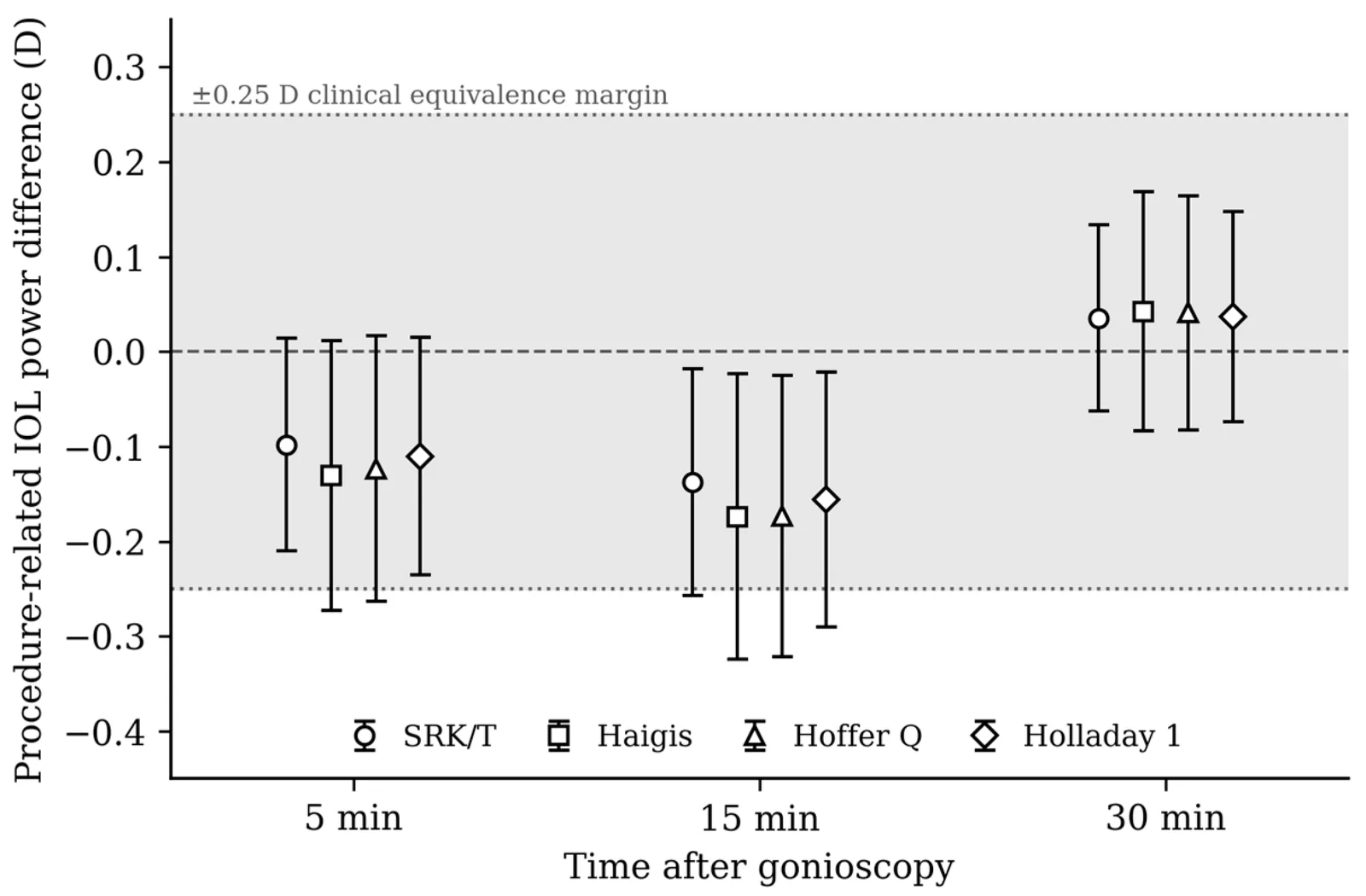
Within-patient difference in differences in continuous calculated IOL power for the SRK/T, Haigis, Hoffer Q and Holladay 1 formulas. The shaded band and dotted lines mark the predefined ±0.25 D clinical relevance margin; error bars indicate 95% confidence intervals. All mean differences remained within the margin at every time point. Filled markers denote the comparisons in which equivalence within ±0.25 D was demonstrated after Holm adjustment across the twelve equivalence tests—30 min for all four formulas and 5 min for SRK/T; open markers denote comparisons in which equivalence was not demonstrated, which is not the same as evidence of a clinically important difference. The equivalence decision rests on the 90% confidence interval used by the two one-sided tests and reported in Table 4, not on the 95% intervals plotted here.

Keratometry barely moved. The largest procedure-related difference at the 2.4-mm zone was +0.107 D at 15 min (95% CI, +0.008 to +0.206; unadjusted *p* = 0.035), and at 3.3 mm, +0.086 D at the same point. For an average eye in this cohort (AL 23.52 mm, Km 43.94 D), the sensitivity of calculated power to corneal power is −1.06 D per diopter of keratometry, so a keratometric change of +0.107 D predicts an IOL power change of approximately −0.11 D. The observed formula outputs are consistent with this: the largest mean IOL differences ranged from −0.14 to −0.17 D at 15 min across the four formulas (Table 4; Figure 2). The keratometric signal and the IOL signal are therefore the same effect expressed in two units, and neither approaches the ±0.25 D margin.

Formal equivalence testing is summarized in Table 4. Against the ±0.25 D margin, and before any adjustment, equivalence was confirmed at 5 and 30 min for all four formulas and at 15 min for SRK/T. Holm adjustment across the twelve tests left five of those conclusions standing: 30 min for SRK/T, Haigis, Hoffer Q and Holladay 1, and 5 min for SRK/T. The rest fell: the three remaining 5 min conclusions, and all four at 15 min. Leave-one-out testing pointed the same way. The unadjusted Haigis and Hoffer Q verdicts at 5 min reversed in 43 and six replicates, and the SRK/T verdict at 15 min in seven; SRK/T and Holladay 1 at 5 min, and all four formulas at 30 min, reversed for no single-patient exclusion. Every point estimate lay inside the margin. The largest was −0.18 D, and the lower 90% confidence limits at 15 min dropped only 0.02 to 0.06 D beyond it. But a point estimate inside a margin is not a demonstration that the true difference lies there, and we do not treat the two as interchangeable. Failure to demonstrate equivalence says something about the precision available with this sample and this number of tests. It is not evidence of a clinically important difference.

### 3.5. Change in the Lens Power That Would Have Been Selected

Because the device stores powers already rounded to the 0.5 D supply grid, a change in the stored value is a change in the lens that would have been ordered. Changes of that kind were common. They were not, however, specific to gonioscopy (Table 5). The selected power changed in 34.4–45.3% of study eyes for SRK/T and 32.8–46.9% for Haigis across the three time points. Untreated fellow control eyes, which underwent no procedure, showed comparable rates of 31.2–43.8% and 32.8–51.6% respectively. In most affected eyes the change was a single 0.5 D step. Pooled across the six formula-by-time-point comparisons, 82.2% of the changes in study eyes and 81.8% of those in control eyes were of one step, and a change of two or more steps occurred in 17.8% of affected study-eye observations against 18.2% of affected control-eye observations. These pooled figures do not apply to any single comparison; the full distribution, cell by cell, is given in Supplementary Table S2.

The procedure-attributable excess never exceeded 15 percentage points and was inconsistent in direction. The only nominally significant paired comparison was Haigis at 15 min, where the change was more frequent in control than in study eyes (51.6% versus 32.8%; *p* = 0.029), the opposite of a procedure effect, and therefore most consistent with sampling variation in a series of underpowered paired tests. Taken together with the mean-level results, these proportions indicate that step-level changes in selected lens power are a property of repeated optical biometry rather than a consequence of gonioscopy.

### 3.6. Sensitivity Analyses

Seven sensitivity analyses follow. The first two concern the handling of the two out-of-range entries; Supplementary Note S1 sets all three handlings side by side, test by test. First, replacing those entries with the reconstructed values 45.42 D and 3.12 mm, rather than treating them as missing, changed no estimate materially: the keratometric DiD at 15 min moved from +0.110 to +0.107 D, the calculated-power differences by no more than 0.006 D, and no equivalence verdict altered, adjusted or unadjusted. Second, excluding both patients from the cohort entirely (*n* = 62 pairs) reproduced the same picture: the CCT peak was +3.27 µm at 5 min, the 15 min calculated-power differences ranged from −0.142 to −0.180 D, and the same five conclusions survived Holm adjustment. Third, restricting the analysis to the 60 patients also reported in the source thesis left every conclusion intact: the CCT peak was +3.27 µm at 5 min and +1.82 µm at 15 min, and equivalence was confirmed in unadjusted testing at 5 and 30 min for all four formulas, with SRK/T at 15 min falling marginally outside the threshold (TOST *p* 0.038 to 0.066) as the sample shrank. Fourth, replacing the nested patient/eye model with a single eye-level random intercept reproduced every interaction test statistic to two decimal places. Fifth, because the corneal response to contact procedures might plausibly differ between younger and older eyes, the per-patient difference-in-differences estimates were regressed on age. No association was found at any time point for CCT (Pearson r = +0.04, +0.11 and −0.01 at 5, 15 and 30 min; all *p* > 0.39) or for calculated IOL power (all |r| < 0.09, all *p* > 0.49), and dichotomizing at the median age of 71 years gave near-identical CCT estimates in the younger and older halves (+3.15 versus +3.13 µm at 5 min; *p* = 0.99). Within the age range studied (46 to 93 years) the transient response did not vary with age. Sixth, the outlier screen described in Section 2.7 flagged 21 individual measurements in 13 patients, most of them white-to-white and axial-length readings that moved and returned within a single session. Excluding these 13 patients entirely (*n* = 51 pairs) left the CCT finding intact (+3.14 µm at 5 min, unchanged; +2.12 µm at 15 min) while the calculated-power differences moved towards zero at every time point: −0.087 D for SRK/T and −0.116 D for Haigis at 15 min, against −0.140 and −0.180 D in the full sample. Two limits govern how far this can be read. The thresholds were defined at the analysis stage rather than in advance, and the screen selected for observations that moved, so removing them makes equivalence easier to declare irrespective of why those observations moved. This analysis therefore shows that the 15 min result depends on a minority of patients; it does not show that the full-sample failure of equivalence was caused by unstable readings rather than by a procedure effect concentrated in those eyes, and we draw no causal conclusion from it. Excluding a fifth of the cohort on a criterion correlated with the outcome also biases the remaining estimate towards the null. Seventh, repeating every primary comparison with the Wilcoxon signed-rank test reproduced every conclusion. In the submitted version, mean keratometry at 15 min was nominally significant on the paired t test and fell marginally short on the rank test; with the two out-of-range entries treated as missing, the two tests now agree (t *p* = 0.033, rank *p* = 0.046). That endpoint does not survive multiplicity adjustment in either analysis and is treated throughout as exploratory. Medians were consistently smaller in magnitude than means for the calculated-power endpoints, indicating that the mean differences are pulled by a minority of eyes rather than reflecting a shift of the whole distribution.

## 4. Discussion

Uncomplicated Goldmann three-mirror gonioscopy produced a CCT signal that was statistically detectable across 64 paired eyes but small enough to fall inside ordinary measurement repeatability. Every procedure-related difference in calculated IOL power was small, at most −0.18 D. The distinction between a small point estimate and demonstrated equivalence matters here. After adjustment across the twelve equivalence tests performed, equivalence within ±0.25 D was demonstrated at 30 min for all four formulas and at 5 min for SRK/T; at 15 min, it was not demonstrated for Haigis, Hoffer Q or Holladay 1. The workflow question raised in the Introduction therefore gets a qualified answer rather than a simple one. The disturbance we measured is small in every comparison, but these data do not establish that it is negligible throughout the first half hour, and we make no recommendation about deferring biometry. That contact examination disturbs the ocular surface is already known. What this study adds is a bound on it, measured against an untreated fellow eye and expressed in the units in which the decision is actually made.

The response peaked at 5 min and had largely resolved by 30. Its origin is less certain. Gonioscopy places viscous coupling gel and topical anesthetic directly on the cornea, and a thin residual layer at the optical interface can be read by the biometer as added thickness while the stroma is untouched. The agent used here was a carbomer 980 gel, which has a longer ocular residence time than hypromellose preparations, so a short-lived residual film remains plausible even though the surface was irrigated after the lens came out. True epithelial or stromal swelling after mild mechanical contact cannot be excluded, and the two may coexist. Our data cannot separate them. The front-loaded time course fits a surface artefact at least as well as edema, and we read the finding as an optical-biometric signal rather than as corneal swelling.

A mean difference of 3.14 µm is about 0.6% of central corneal thickness. It sits inside the ±6.2 µm band within which the untreated fellow eyes of these same patients varied over the same half hour. No single measurement can be attributed to the procedure on that basis, and no decision resting on CCT in one eye would change: pachymetric correction of applanation tonometry, for instance, shifts by well under 0.1 mmHg [24,25,26]. The signal is visible only because it is averaged over 64 pairs. Its value is mainly negative, as an upper bound on how much this procedure disturbs the cornea.

That bound also answers whether a formula using CCT as an input would have behaved differently. In the vergence structure these formulas share, calculated power changes by roughly 1.88 D per millimeter of effective lens position for an average eye in this cohort. A CCT change of 3.14 µm is 0.00314 mm. Transmitted one-to-one into the effective-lens-position estimate, which is far more than any published formula does, it would move calculated power by 0.006 D. We could not test such a formula directly: Kane is not published in closed form, offers no batch interface, and needs the lens thickness, which the AL-Scan does not measure. That is a real gap in the formula set. The arithmetic above suggests it is not a gap in the conclusion.

Our findings sit somewhat apart from the earlier gonioscopy literature. George et al. measured keratometry and topography after gonioscopy, saw transient steepening in the early minutes, and warned against keratometry immediately afterwards where curvature is the quantity of interest [18]. Here, keratometry moved by at most 0.11 D and calculated power by at most 0.18 D, and it was CCT that carried the clearest signal. The older population, the device, the standardized three-mirror technique and the within-subject control could all contribute to the difference.

One point needs correcting from the version we submitted, which reported a dissociation between Haigis and SRK/T. Both draw on the same axial length and keratometry, and Haigis adds ACD, so a real curvature or depth change should move them together. In the rounded outputs, it did not. Recomputation from continuous values showed why: quantization onto the 0.5 D grid turns a sub-step systematic difference into an apparently discrete one and does so unevenly for formulas whose unrounded outputs differ by hundredths of a diopter. The corrected picture is a small, coherent, formula-independent effect of −0.14 to −0.17 D at 15 min.

The untreated fellow eye earned its place in the design. Biometry fluctuated in eyes that never underwent gonioscopy, and the paired estimator measured procedure-related change against exactly that background. It is not a randomized control though. Study eyes started with lower BCVA because allocation followed clinical need, and denser cataracts may have made their biometry intrinsically noisier. Two further limits deserve stating. Only one pre-procedure measurement was taken, so the parallel-trends assumption behind the estimator could not be checked; a repeated baseline should be built into any replication. And the fellow eye is contemporaneous but not equivalent. What the design does establish is that any procedure-related change is small relative to the background variation seen in eyes measured on the same schedule without the procedure.

The individual-eye analysis answers something the means cannot. Between a third and a half of eyes shifted by a full 0.5 D step in the power that would have been ordered, from one measurement to the next within a single session. Untreated control eyes did the same, sometimes more often. This is ordinary test–retest behavior on a supply grid, not a consequence of gonioscopy. Two things follow: gonioscopy does not raise the odds that a given eye’s recommended power will change, and single-measurement IOL power scatters enough on its own to argue for confirmatory or averaged biometry in borderline eyes.

Several limitations apply. The most important one for how this report should be read is that no prospective analysis plan or sample size calculation existed and the endpoint hierarchy was set during analysis, so every *p* value here is exploratory, the equivalence conclusions included. The ±0.25 D margin follows from the 0.5 D supply grid rather than from the data, but that does not make the study confirmatory. Retaining CCT rather than keratometry as the nominal primary endpoint is a label without inferential weight: both are reported with the same estimator and the same multiplicity treatment, and keratometry is the one carrying the mechanistic link to calculated power. Allocation followed clinical indication rather than randomization, baseline BCVA differed between groups, and a single examiner performed both gonioscopy and biometry without masking. Random slopes and serial residual covariance structures were not modeled. Twelve TOST analyses were run, and although Holm adjustment across the family is now applied, no principal comparison was defined in advance and the adjusted conclusions remain exploratory rather than confirmatory; three of the four 5 min verdicts and all four 15 min verdicts did not survive adjustment, and two of the unadjusted 5 min verdicts reversed under leave-one-out testing. Twenty-six of the 33 difference distributions were non-normal, with heavy tails contributed by a minority of eyes; the rank-based check reproduced every conclusion but one. The outlier screen was defined after the data had been seen and is reported as a sensitivity analysis only; it cannot distinguish measurement instability from a procedure effect concentrated in a minority of eyes.

Finally, the dataset is roughly a decade old and comes from one center, one Goldmann lens, one partial-coherence biometer, a 30 min window and a formula set that predates current suites. Contemporary swept-source devices resolve the ocular surface differently and might not reproduce a signal of this size in either direction. Different contact lenses and eyes with substantial ocular surface disease were not tested. We present these findings as period- and device-specific and would regard replication on a modern biometer as necessary before they are generalized [2,27].

## 5. Conclusions

In this 2015–2016 AL-Scan cohort, uncomplicated Goldmann three-mirror gonioscopy produced a small, transient rise in measured central corneal thickness. It peaked at 5 min, was gone by 30, and never left the range of ordinary short-term measurement variability. Axial length, anterior chamber depth, white-to-white diameter, pupil diameter and keratometry were clinically unchanged. Every procedure-related difference in calculated IOL power was small: at most −0.18 D, inside the ±0.25 D margin and below the 0.5 D supply interval. Formal equivalence is the harder claim, and after adjustment across the twelve tests performed, it was demonstrated at 30 min for all four formulas and at 5 min for SRK/T. At 15 min it was not demonstrated for Haigis, Hoffer Q or Holladay 1. We therefore report a small observed effect rather than established equivalence across the observation window and make no recommendation about whether biometry should be deferred. The lens power that would actually have been ordered changed just as often in eyes that had no gonioscopy at all. That locates most of the step-level variability in the measurement rather than in the procedure. Where pachymetry is the objective, taking it before contact gonioscopy remains the simpler default. All of this is exploratory. It belongs to the device, lens and formulas of the study period and should be checked on modern biometers before it is generalized.

## Figures and Tables

**Table 1:** Baseline characteristics of study and fellow control eyes.

Parameter	Study Eyes (*n* = 64)	Control Eyes (*n* = 64)	Paired *p*
BCVA (decimal Snellen)	0.313 ± 0.169	0.602 ± 0.276	<0.001
CCT (µm)	523.5 ± 28.5	523.6 ± 28.4	0.945
Mean K, 2.4 mm (D)	43.87 ± 1.67	44.00 ± 1.63	0.111
Mean K, 3.3 mm (D)	43.83 ± 1.66	43.92 ± 1.62	0.224
Axial length (mm)	23.54 ± 1.36	23.50 ± 1.44	0.228
ACD (mm)	3.30 ± 0.42	3.23 ± 0.38	0.035
WTW (mm)	11.68 ± 0.62	11.79 ± 0.44	0.040
Pupil diameter (mm)	4.74 ± 0.98	4.68 ± 0.87	0.372
SRK/T power (D)	20.52 ± 3.30	20.54 ± 3.68	0.918
Haigis power (D)	20.61 ± 3.22	20.58 ± 3.57	0.838

Values are mean ± standard deviation. *p* values are from paired *t* tests comparing the two eyes of the same patient. Mean age 69.44 ± 9.79 years; female/male 32/32; right/left study eyes 35/29; Shaffer grade in study eyes, median 3 (range 2–4). BCVA was used for baseline description only and was not modeled.

**Table 2:** Group-by-time interaction results from nested patient/eye mixed-effects models.

Endpoint Family	Parameter	χ^2^	df	*p* Value	Holm-Adjusted *p*
Primary	CCT (µm)	29.95	3	<0.001	Not applicable
Secondary	Mean K, 2.4 mm (D)	9.97	3	0.019	0.094
Secondary	Mean K, 3.3 mm (D)	12.22	3	0.007	0.045
Secondary	Axial length (mm)	3.79	3	0.285	0.856
Secondary	ACD (mm)	5.29	3	0.152	0.607
Secondary	WTW (mm)	0.37	3	0.947	1.000
Secondary	Pupil diameter (mm)	1.53	3	0.675	1.000
Secondary	SRK/T power (D)	13.06	3	0.005	0.045
Secondary	Haigis power (D)	12.99	3	0.005	0.045
Secondary	Hoffer Q power (D)	12.98	3	0.005	0.045
Secondary	Holladay 1 power (D)	12.87	3	0.005	0.045

Likelihood-ratio tests compare models with and without the group-by-time interaction. Holm adjustment was applied across the ten secondary endpoints only. CCT was designated primary during analysis, not prospectively (Section 2.3).

**Table 3:** Within-patient paired difference in differences for central corneal thickness.

Time Point	DiD (µm)	SE	95% CI	*p* Value
5 min	+3.14	0.60	+1.94 to +4.35	<0.001
15 min	+1.66	0.66	+0.33 to +2.98	0.015
30 min	+0.45	0.68	−0.90 to +1.80	0.505

Difference in differences represents additional post-baseline change in study eyes relative to the untreated fellow control eye of the same patient; *n* = 64 patient pairs. Estimates are unadjusted for multiplicity and are exploratory.

**Table 4:** Within-patient difference in differences and equivalence testing for continuous calculated IOL power (±0.25 D margin).

Formula	Time	Mean Difference (D)	90% CI	TOST *p* (Unadj.|Holm)	Equivalence (Holm)
SRK/T	5 min	−0.098	−0.192 to −0.005	0.004|0.035	Confirmed
SRK/T	15 min (*n* = 63)	−0.140	−0.242 to −0.039	0.038|0.227	Not confirmed
SRK/T	30 min	+0.035	−0.047 to +0.117	<0.001|<0.001	Confirmed
Haigis	5 min	−0.131	−0.249 to −0.012	0.049|0.227	Not confirmed
Haigis	15 min (*n* = 62)	−0.180	−0.309 to −0.050	0.183|0.334	Not confirmed
Haigis	30 min	+0.042	−0.063 to +0.148	0.001|0.007	Confirmed
Hoffer Q	5 min	−0.124	−0.241 to −0.007	0.038|0.227	Not confirmed
Hoffer Q	15 min (*n* = 63)	−0.177	−0.302 to −0.051	0.167|0.334	Not confirmed
Hoffer Q	30 min	+0.041	−0.062 to +0.144	0.001|0.006	Confirmed
Holladay 1	5 min	−0.110	−0.215 to −0.005	0.015|0.103	Not confirmed
Holladay 1	15 min (*n* = 63)	−0.159	−0.273 to −0.045	0.093|0.280	Not confirmed
Holladay 1	30 min	+0.037	−0.056 to +0.130	<0.001|0.002	Confirmed

Equivalence within ±0.25 D was declared when the 90% confidence interval lay entirely inside the margin. The unadjusted TOST *p* is the larger of the two one-sided *p* values; the adjusted value is Holm-corrected across the full family of twelve tests, and the Equivalence column reports the adjusted verdict. *n* varies at 15 min because two erroneous entries are treated as missing (Section 2.7). IOL power was computed as a continuous variable with A = 118.70. “Not confirmed” indicates that equivalence was not demonstrated, not that a clinically meaningful difference was shown. All equivalence conclusions are exploratory.

**Table 5:** Proportion of eyes in which the selected 0.5-D IOL power changed from baseline.

Formula	Time	Study Eyes *n* (%)	Control Eyes *n* (%)	Excess (pp)	McNemar *p*
SRK/T	5 min	29 (45.3)	20 (31.2)	+14.1	0.108
SRK/T	15 min	22 (34.4)	28 (43.8)	−9.4	0.345
SRK/T	30 min	28 (43.8)	21 (32.8)	+10.9	0.265
Haigis	5 min	30 (46.9)	25 (39.1)	+7.8	0.424
Haigis	15 min	21 (32.8)	33 (51.6)	−18.8	0.029
Haigis	30 min	27 (42.2)	21 (32.8)	+9.4	0.307

Computed from device-stored powers, which are rounded to the 0.5 D supply grid. Among the observations in which the power changed, 82.2% in study eyes and 81.8% in control eyes changed by a single step, the remainder by two or more; the full distribution is given in Supplementary Table S2. Excess is the study-minus-control difference in percentage points (pp); *n* = 64 per group.

## Data Availability

The anonymized participant-level dataset (Dataset S1) and the complete analysis code (Script S1) are provided as Supplementary Materials. Dataset S1 contains an analysis_dataset sheet, used for every estimate reported here, and an as_recorded sheet preserving every value exactly as originally entered, including the two out-of-range entries discussed in Section 2.7, so that the handling decision can be inspected directly. Script S1 reads Dataset S1 and reproduces every estimate reported in Table 1, Table 2, Table 3, Table 4 and Table 5, Figure 1 and Figure 2, Supplementary Tables S1 and S2 and Supplementary Note S1, including all seven sensitivity analyses, the multiplicity adjustment and the device-configuration check. The original AL-Scan printouts and paper data-collection forms are held in the hospital archive rather than by the authors personally; they are retrievable through the institution’s records procedure and will be supplied to the Editorial Office on request. No value in the analysis was reconstructed, because none of the recorded entries had been checked against a source document at the time of analysis. The residency thesis drawn from the same source records for 60 of the 64 patients, including both patients with out-of-range entries, is publicly deposited in the Turkish Council of Higher Education National Thesis Centre [20].

## Supplementary Materials

The following supporting information can be downloaded at: https://www.mdpi.com/article/10.3390/medicina62081576/s1; Table S1: Full exploratory pairwise difference in differences for all parameters, with Holm-adjusted *p* values; Table S2: Distribution of change in the selected 0.5 D IOL power, by formula, time point and group; Note S1: sensitivity of every reported conclusion to the handling of the two out-of-range entries; Dataset S1: anonymized participant-level dataset, with an as_recorded sheet preserving every value as originally entered; Script S1: complete analysis code.
