# Supplementary material for: Transient Corneal Response After Goldmann Three-Mirror Gonioscopy and Its Limited Effect on Optical Biometry and Calculated Intraocular Lens Power: A Prospective Within-Subject Controlled Observational Study
Source: Medicina (Kaunas). 2026 Aug 17;62(8):1576. doi: 10.3390/medicina62081576 (PMC13515283; doi:10.3390/medicina62081576)
Supplement: Supplementary file 1 [file medicina-62-01576-s001.zip › medicina-4450377-supplementary.pdf]

Supplementary Materials

Transient Corneal Response After Goldmann Three-Mirror Gonioscopy and Its Limited Effect on Optical Biometry and Calculated Intraocular Lens Power: A Prospective Within-Subject Controlled Observational Study

Medicina — manuscript medicina-4450377

Contents

Item	Description
Table S1	Full exploratory pairwise difference-in-differences for all parameters, with Holm-adjusted P values.
Table S2	Distribution of change in the selected 0.5-D IOL power, by formula, time point and group.
Note S1	Sensitivity of every reported conclusion to the handling of the two out-of-range entries.
Dataset S1	Anonymized participant-level dataset. Field definitions are given in Section 4 below; the machine-readable workbook is supplied as a separate file, medicina-4450377_DatasetS1.xlsx.
Script S1	Complete analysis code, listed in full in Section 5 below and supplied as a separate file, medicina-4450377_ScriptS1.py.

1. Table S1

Full exploratory pairwise difference-in-differences for all parameters.

Parameter	Time	n	DiD	SE	95% CI	P value	Holm P
CCT (μm)	5 min	64	+3.141	0.603	+1.936 to +4.345	<0.001	<0.001
CCT (μm)	15 min	64	+1.656	0.665	+0.328 to +2.985	0.015	0.492
CCT (μm)	30 min	64	+0.453	0.676	−0.897 to +1.803	0.505	1.000
Mean K, 2.4 mm (D)	5 min	64	+0.083	0.050	−0.017 to +0.183	0.102	1.000
Mean K, 2.4 mm (D)	15 min	63	+0.110	0.050	+0.009 to +0.210	0.033	0.868
Mean K, 2.4 mm (D)	30 min	64	−0.021	0.045	−0.110 to +0.068	0.645	1.000
Mean K, 3.3 mm (D)	5 min	64	−0.012	0.041	−0.093 to +0.069	0.775	1.000
Mean K, 3.3 mm (D)	15 min	64	+0.086	0.038	+0.010 to +0.162	0.026	0.716
Mean K, 3.3 mm (D)	30 min	64	−0.049	0.035	−0.119 to +0.021	0.169	1.000
Axial length (mm)	5 min	64	+0.004	0.006	−0.007 to +0.015	0.455	1.000
Axial length (mm)	15 min	64	+0.009	0.008	−0.007 to +0.025	0.288	1.000
Axial length (mm)	30 min	64	−0.003	0.005	−0.014 to +0.008	0.568	1.000
ACD (mm)	5 min	64	−0.015	0.012	−0.039 to +0.008	0.197	1.000
ACD (mm)	15 min	63	+0.000	0.003	−0.006 to +0.007	0.889	1.000
ACD (mm)	30 min	64	+0.003	0.005	−0.006 to +0.013	0.491	1.000
WTW (mm)	5 min	64	+0.000	0.042	−0.084 to +0.084	1.000	1.000
WTW (mm)	15 min	64	−0.012	0.043	−0.098 to +0.073	0.772	1.000
WTW (mm)	30 min	64	+0.013	0.035	−0.057 to +0.082	0.721	1.000
Pupil diameter (mm)	5 min	64	+0.047	0.063	−0.079 to +0.172	0.458	1.000
Pupil diameter (mm)	15 min	64	+0.052	0.077	−0.103 to +0.206	0.507	1.000
Pupil diameter (mm)	30 min	64	−0.039	0.068	−0.175 to +0.097	0.567	1.000
SRK/T (D)	5 min	64	−0.098	0.056	−0.210 to +0.014	0.085	1.000
SRK/T (D)	15 min	63	−0.140	0.061	−0.262 to −0.019	0.024	0.695
SRK/T (D)	30 min	64	+0.035	0.049	−0.063 to +0.133	0.474	1.000
Haigis (D)	5 min	64	−0.131	0.071	−0.273 to +0.011	0.071	1.000
Haigis (D)	15 min	62	−0.180	0.078	−0.335 to −0.025	0.024	0.695
Haigis (D)	30 min	64	+0.042	0.063	−0.084 to +0.168	0.505	1.000
Hoffer Q (D)	5 min	64	−0.124	0.070	−0.264 to +0.016	0.083	1.000
Hoffer Q (D)	15 min	63	−0.177	0.075	−0.327 to −0.027	0.022	0.676
Hoffer Q (D)	30 min	64	+0.041	0.062	−0.082 to +0.164	0.510	1.000
Holladay 1 (D)	5 min	64	−0.110	0.063	−0.235 to +0.015	0.084	1.000

Parameter	Time	n	DiD	SE	95% CI	P value	Holm P
Holladay 1 (D)	15 min	63	−0.159	0.068	−0.295 to −0.023	0.023	0.688
Holladay 1 (D)	30 min	64	+0.037	0.056	−0.074 to +0.148	0.506	1.000

Difference-in-differences estimates represent additional post-baseline change in study eyes relative to the untreated fellow control eye of the same patient, computed by within-patient paired analysis. Calculated IOL powers are continuous, unrounded values obtained with A = 118.70; keratometry is the mean of K1 and K2 at the stated optical zone. n falls below 64 at 15 minutes for the parameters affected by the two out-of-range entries, which are treated as missing rather than reconstructed (Section 2.7 of the manuscript); a patient contributes to a comparison only where every value entering it is present. Holm-adjusted P values were calculated across this exploratory pairwise family of 33 comparisons and are provided for completeness. As stated in the main text, these pairwise estimates are exploratory and the study was not prospectively powered. Formal equivalence testing for the four IOL formulas, with Holm adjustment across its own family of twelve tests, is reported in Table 4 of the main text.

2. Table S2

Distribution of change in the selected 0.5-D IOL power, by formula, time point and group.

Formula	Time	Study eyes: changed n (%)	Study: 1 step	Study: ≥2 steps	Control eyes: changed n (%)	Control: 1 step	Control: ≥2 steps
SRK/T	5 min	29 (45.3)	25	4	20 (31.2)	17	3
SRK/T	15 min	22 (34.4)	19	3	28 (43.8)	23	5
SRK/T	30 min	28 (43.8)	25	3	21 (32.8)	18	3
Haigis	5 min	30 (46.9)	23	7	25 (39.1)	23	2
Haigis	15 min	21 (32.8)	16	5	33 (51.6)	24	9
Haigis	30 min	27 (42.2)	21	6	21 (32.8)	16	5
Pooled	all	157 / 384	129 (82.2%)	28 (17.8%)	148 / 384	121 (81.8%)	27 (18.2%)

Computed from the device-stored powers, which are already rounded to the 0.5-D interval on which lenses are supplied, so that a change in the stored value is a change in the lens that would have been ordered. "Changed" counts eyes in which the stored power at the given time point differs from the same eye's baseline value; step counts are of the eyes that changed. n = 64 eyes per group per cell. In the pooled row, percentages are of the number of changed observations rather than of 64. The pooled figures quoted in Section 3.5 of the manuscript are these values and do not apply to any single formula-by-time-point comparison taken alone.

3. Note S1

Sensitivity of every reported conclusion to the handling of two out-of-range entries.

3.1 The two entries

Two isolated entries in the dataset fall outside the range the NIDEK AL-Scan can return. Both are in control eyes at the 15-minute time point, and in each the leading digit is absent:

- Participant P012, control eye: anterior chamber depth recorded as 0.12 mm, against 3.13, 3.20 and 3.12 mm at the other three time points in the same eye.
- Participant P015, control eye: K1 at the 2.4-mm zone recorded as 5.42 D, against 45.36, 45.30 and 45.24 D at the other three time points in the same eye.

That these entries are not measurements follows from the instrument specification. What the true values were does not, and no source-document check has been made. The analysis reported in the manuscript therefore reconstructs nothing: both entries are treated as missing, and each patient contributes to a comparison only where every value entering it is present. This note sets out what would change under the two alternative handlings. Nothing of consequence does, and that is the point: the choice is not load-bearing for any conclusion in the paper.

3.2 The three handlings

Handling	What is done	Patients	n at 15 min
A — missing (used in the manuscript)	Both entries set to missing. No value imputed. A patient contributes to a comparison only where every value entering it is present.	64	63 for mean K, SRK/T, Hoffer Q and Holladay 1; 62 for Haigis, which uses measured ACD
B — reconstructed	The absent leading digit is restored: 3.12 mm and 45.42 D. This was the handling in the originally submitted version and has been withdrawn.	64	64 throughout
C — excluded	Both patients are removed from the cohort entirely, at every time point.	62	62 throughout

Handling A is the most conservative of the three. It discards the two erroneous entries without asserting a replacement and without discarding the 126 valid measurements those two patients also contributed. Handling C is the most wasteful: it removes two complete patients because one measurement in each was mis-transcribed.

3.3 Central corneal thickness and keratometry

Endpoint	A — treated as missing	B — reconstructed values	C — both patients excluded
CCT (µm), 5 min	+3.141 (n = 64, P = <0.001)	+3.141 (n = 64, P = <0.001)	+3.274 (n = 62, P = <0.001)
CCT (µm), 15 min	+1.656 (n = 64, P = 0.015)	+1.656 (n = 64, P = 0.015)	+1.581 (n = 62, P = 0.024)
CCT (µm), 30 min	+0.453 (n = 64, P = 0.505)	+0.453 (n = 64, P = 0.505)	+0.419 (n = 62, P = 0.547)
Mean K 2.4 mm (D), 5 min	+0.0830 (n = 64, P = 0.102)	+0.0830 (n = 64, P = 0.102)	+0.0858 (n = 62, P = 0.101)
Mean K 2.4 mm (D), 15 min	+0.1096 (n = 63, P = 0.033)	+0.1069 (n = 64, P = 0.035)	+0.1098 (n = 62, P = 0.036)
Mean K 2.4 mm (D), 30 min	−0.0206 (n = 64, P = 0.645)	−0.0206 (n = 64, P = 0.645)	−0.0196 (n = 62, P = 0.670)

Neither erroneous entry is a CCT value, so handlings A and B give identical CCT estimates. Handling C differs only because two patients are gone: the 5-minute peak moves from +3.14 to +3.27 µm, which is smaller than the ±6.2 µm short-term repeatability observed in untreated control eyes. Every significance verdict is the same under all three.

3.4 Calculated IOL power: all twelve equivalence tests

Difference-in-differences against the ±0.25 D margin, tested by two one-sided tests, with Holm adjustment across the family of twelve. The verdict column reports the adjusted result.

Formula	Time	A: DiD	A: P raw/Holm	B: DiD	B: P raw/Holm	C: DiD	C: P raw/Holm	Verdict (all three)
SRK/T	5 min	−0.098 (n=64)	0.004 / 0.035	−0.098 (n=64)	0.004 / 0.035	−0.101 (n=62)	0.006 / 0.049	Confirmed
SRK/T	15 min	−0.140 (n=63)	0.038 / 0.227	−0.138 (n=64)	0.032 / 0.194	−0.142 (n=62)	0.042 / 0.254	Not confirmed
SRK/T	30 min	+0.035 (n=64)	<0.001 / <0.001	+0.035 (n=64)	<0.001 / <0.001	+0.034 (n=62)	<0.001 / <0.001	Confirmed
Haigis	5 min	−0.131 (n=64)	0.049 / 0.227	−0.131 (n=64)	0.049 / 0.197	−0.134 (n=62)	0.060 / 0.254	Not confirmed
Haigis	15 min	−0.180 (n=62)	0.183 / 0.334	−0.174 (n=64)	0.158 / 0.305	−0.180 (n=62)	0.183 / 0.354	Not confirmed
Haigis	30 min	+0.042 (n=64)	0.001 / 0.007	+0.042 (n=64)	0.001 / 0.007	+0.040 (n=62)	0.001 / 0.009	Confirmed
Hoffer Q	5 min	−0.124 (n=64)	0.038 / 0.227	−0.124 (n=64)	0.038 / 0.194	−0.127 (n=62)	0.047 / 0.254	Not confirmed
Hoffer Q	15 min	−0.177 (n=63)	0.167 / 0.334	−0.173 (n=64)	0.152 / 0.305	−0.179 (n=62)	0.177 / 0.354	Not confirmed
Hoffer Q	30 min	+0.041 (n=64)	0.001 / 0.006	+0.041 (n=64)	0.001 / 0.006	+0.039 (n=62)	0.001 / 0.007	Confirmed
Holladay 1	5 min	−0.110 (n=64)	0.015 / 0.103	−0.110 (n=64)	0.015 / 0.103	−0.113 (n=62)	0.019 / 0.134	Not confirmed
Holladay 1	15 min	−0.159 (n=63)	0.093 / 0.280	−0.156 (n=64)	0.083 / 0.249	−0.161 (n=62)	0.101 / 0.303	Not confirmed
Holladay 1	30 min	+0.037 (n=64)	<0.001 / 0.002	+0.037 (n=64)	<0.001 / 0.002	+0.035 (n=62)	<0.001 / 0.002	Confirmed

All twelve equivalence verdicts are identical under the three handlings. Equivalence within ±0.25 D is demonstrated, after Holm adjustment, at 30 minutes for SRK/T, Haigis, Hoffer Q and Holladay 1, and at 5 minutes for SRK/T. It is not demonstrated for the remaining seven comparisons. No handling moves any comparison across the threshold in either direction.

3.5 How far the estimates move

Quantity	Largest difference across the three handlings	For scale
Calculated IOL power (any formula, any time point)	0.006 D (Haigis at 15 minutes: −0.180 D under A and C, −0.174 D under B)	The supply grid is 0.5 D. The equivalence margin is 0.25 D.
Mean keratometry, 2.4 mm zone	0.003 D	Device keratometric repeatability is an order of magnitude larger.
Central corneal thickness	0.13 µm (handling C only)	Short-term repeatability in untreated control eyes is ±6.2 µm.
Equivalence verdicts (12 tests)	None. All twelve agree under all three handlings.	—

3.6 What follows

The largest movement any conclusion sustains across the three handlings is 0.006 D in a single point estimate — roughly one eightieth of the interval on which intraocular lenses are supplied, and one fortieth of the equivalence margin. No verdict changes, no significance conclusion changes, and no statement in the manuscript would have to be rewritten under any of the three.

This matters for how the two entries should be read. A reader who accepts the reconstruction, a reader who rejects it, and a reader who would rather see both patients removed all arrive at the same paper. The manuscript adopts handling A because it is the one that asserts least: it uses no value that cannot be traced to the recorded dataset. Should the original device printouts be examined and the reconstructed values confirmed, handling B would become available, but nothing in the results would move, and we would not change the analysis on that account.

4. Dataset S1

Anonymized participant-level dataset.

The workbook is supplied as a separate machine-readable file (medicina-4450377_DatasetS1.xlsx) so that Script S1 can be run against it directly. It contains 64 patients and 128 eyes across three sheets:

- analysis_dataset — the data from which every reported estimate is computed. The two out-of-range entries are blank here, in line with handling A above; no value has been imputed in their place.
- as_recorded — every value exactly as originally entered, including both out-of-range entries in their original form, so that the handling decision can be inspected rather than taken on trust.
- data_dictionary — field definitions, reproduced below.

Field definitions

Field	Description
participant_id	study participant code (hospital identifiers removed)
eye	study eye (gonioscopy) or untreated fellow control
laterality	laterality
age_years	age at enrolment, years
sex	sex
shaffer_grade	Shaffer grade, study eye only
bcva_decimal	best-corrected visual acuity, decimal Snellen
K1_2.4mm_t0 / _t5 / _t15 / _t30	t0 = pre-gonioscopy baseline; t5/t15/t30 = minutes after removal of the gonioscopy lens; each value is the mean of three acquisitions
K2_2.4mm_t0 / _t5 / _t15 / _t30	t0 = pre-gonioscopy baseline; t5/t15/t30 = minutes after removal of the gonioscopy lens; each value is the mean of three acquisitions
K1_3.3mm_t0 / _t5 / _t15 / _t30	t0 = pre-gonioscopy baseline; t5/t15/t30 = minutes after removal of the gonioscopy lens; each value is the mean of three acquisitions
K2_3.3mm_t0 / _t5 / _t15 / _t30	t0 = pre-gonioscopy baseline; t5/t15/t30 = minutes after removal of the gonioscopy lens; each value is the mean of three acquisitions
CCT_um_t0 / _t5 / _t15 / _t30	t0 = pre-gonioscopy baseline; t5/t15/t30 = minutes after removal of the gonioscopy lens; each value is the mean of three acquisitions
WTW_mm_t0 / _t5 / _t15 / _t30	t0 = pre-gonioscopy baseline; t5/t15/t30 = minutes after removal of the gonioscopy lens; each value is the mean of three acquisitions
pupil_mm_t0 / _t5 / _t15 / _t30	t0 = pre-gonioscopy baseline; t5/t15/t30 = minutes after removal of the gonioscopy lens; each value is the mean of three acquisitions
ACD_mm_t0 / _t5 / _t15 / _t30	t0 = pre-gonioscopy baseline; t5/t15/t30 = minutes after removal of the gonioscopy lens; each value is the mean of three acquisitions
AL_mm_t0 / _t5 / _t15 / _t30	t0 = pre-gonioscopy baseline; t5/t15/t30 = minutes after removal of the gonioscopy lens; each value is the mean of three acquisitions

Field	Description
SRKT_device_D_t0 / _t5 / _t15 / _t30	t0 = pre-gonioscopy baseline; t5/t15/t30 = minutes after removal of the gonioscopy lens; each value is the mean of three acquisitions
Haigis_device_D_t0 / _t5 / _t15 / _t30	t0 = pre-gonioscopy baseline; t5/t15/t30 = minutes after removal of the gonioscopy lens; each value is the mean of three acquisitions
data_correction_applied	documented transcription corrections

For the four measurement time points, t0 is the pre-gonioscopy baseline and t5, t15 and t30 are minutes after removal of the gonioscopy lens. Each value is the mean of three acquisitions. Hospital identifiers have been removed and participants are referred to by study code only.
